# Using Thermal Interface Resistance for Noninvasive Operando Mapping of Buried Interfacial Lithium Morphology in Solid-State Batteries

**DOI:** 10.1021/acsami.2c23038

**Published:** 2023-03-23

**Authors:** Divya Chalise, Robert Jonson, Joseph Schaadt, Pallab Barai, Yuqiang Zeng, Sumanjeet Kaur, Sean D. Lubner, Venkat Srinivasan, Michael C. Tucker, Ravi S. Prasher

**Affiliations:** † Department of Mechanical Engineering, University of California, Berkeley, Berkeley, California 94720, United States; ‡ Energy Technologies Area, Lawrence Berkeley National Lab, 1 Cyclotron Road, Berkeley, California 94720, United States; § Argonne National Laboratory, Lemont, Illinois 60439, United States; ∥ Department of Mechanical Engineering, Boston University, Boston, Massachusetts 02215, United States

**Keywords:** thermal wave sensing, solid-state batteries, interface morphology, lithium metal, operando characterization

## Abstract

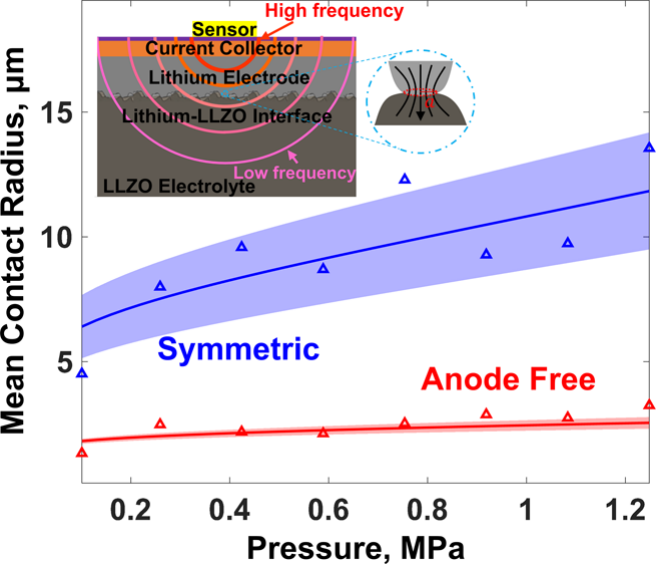

The lithium metal–solid-state electrolyte interface plays a critical role in the performance of solid-state batteries. However, operando characterization of the buried interface morphology in solid-state cells is particularly difficult because of the lack of direct optical access. Destructive techniques that require isolating the interface inadvertently modify the interface and cannot be used for operando monitoring. In this work, we introduce the concept of thermal wave sensing using modified 3ω sensors that are attached to the outside of the lithium metal–solid-state cells to noninvasively probe the morphology of the lithium metal–electrolyte interface. We show that the thermal interface resistance measured by the 3ω sensors relates directly to the physical morphology of the interface and demonstrates that 3ω thermal wave sensing can be used for noninvasive operando monitoring the morphology evolution of the lithium metal–solid-state electrolyte interface.

## Introduction

Lithium metal is widely considered as one of the most promising candidates for next-generation battery anodes, particularly due to its high theoretical capacity (3860 mAh/g) and low reduction potential (−3.04 V vs standard hydrogen electrode (SHE)).^1−4^ However, traditional approaches to using lithium metal anode with liquid electrolyte face significant challenges such as dendrite formation at high current densities and unstable solid–electrolyte interphase (SEI).^2,5^ The lithium metal anode in conjunction with solid-state electrolytes (SSE) is seen as a viable alternative, mainly because a solid electrolyte can potentially act as a physical barrier to dendrite propagation.^6−8^

Among the solid electrolytes, garnet-type electrolyte Li_7_La_3_Zr_2_O_12_ (LLZO) is considered a promising candidate because of high ionic conductivity, large electrochemical stability window, and stability against lithium metal.^9−11^ Recent works have shown that the ionic conductivity of cubic LLZO can reach up to 10^–4^ to 10^–3^ S/cm, which is comparable to that of liquid electrolytes.^12,13^ However, the lithium metal–LLZO interface has prevalent problems.^14,15^ Dendrite propagation along the grain boundaries^11,16^ as well as within a single crystal^17^ has been observed in LLZO electrolyte. Additionally, because of uneven plating and stripping during cycling, the interface between lithium metal and LLZO can develop voids over time, leading to contact loss and a higher cell overpotential and an increased localized current density which can cause dendrite growth.^14,18,19^

Theoretical models based on contact mechanics^20−22^ have been proposed to explain evolution of the interface considering external factors such as the current density and the stack pressure. However, these models have not been directly verified. Various in situ methods such as scanning electron microscopy (SEM),^23,24^ scanning transmission electron microscopy (STEM),^25^ cryo-transmission electron microscopy (cryo-TEM),^26,27^ and X-ray photoelectron spectroscopy (XPS)^13^ have revealed mechanisms of lithium deposition and growth and interface evolution in solid-state electrolytes. However, these methods require isolating the interface, which inadvertently changes the interface and can affect the mechanisms studied. Tomography-based approaches such as X-ray tomography^19^ and magnetic resonance imaging (MRI)^28^ require a highly specialized setup and complicated analysis,^28^ limiting the ease of use and restricting its applicability. Among the global operando techniques, electrochemical impedance spectroscopy (EIS) has been widely used to study the Li–SSE interface.^12,29−31^ A significant problem with EIS, however, is that the interface resistance obtained from EIS is affected by the electrode kinetics,^32,33^ the physical morphology/adhesion of lithium at the interface,^12^ and the presence of surface contaminants,^13^ and determining the contributions of each of these individual effects presents major challenges. Additionally, EIS cannot provide spatial information as it is difficult to attribute specific features to a particular interface.

Thermal wave sensing is based on the 3ω method, which is commonly used for measuring thermal conductivity and thermal interface resistance.^34−37^ Thermal wave sensing has also been used for less typical applications such as fouling sensing,^38^ sedimentation detection,^39^ and determination of gas composition.^40^ More recently, we have shown that an extension of the method can be used for operando determination of thermal interface resistance and the lithium distribution across a battery electrode.^41,42^ In this work, we combine the theory of thermal interface resistance at a metal/nonmetal interface based on viscoelastic deformation of metal at the interface with operando 3ω measurements to develop a method to directly extract the morphological information on the lithium metal–LLZO interface. These findings are verified with ex-situ profilometry and SEM. We also show that the interface morphology information extracted from thermal wave sensing cannot be obtained directly from EIS. Unlike the EIS, which is sensitive to the multiple factors that determine the electrochemical interface resistance, the thermal wave sensing method is only sensitive to the physical morphology of the interface and therefore provides a method to deconvolute the individual factors contributing to the electrochemical interface resistance.

## Morphology from the 3ω Thermal Contact Resistance Measurement

**Figure 1:**
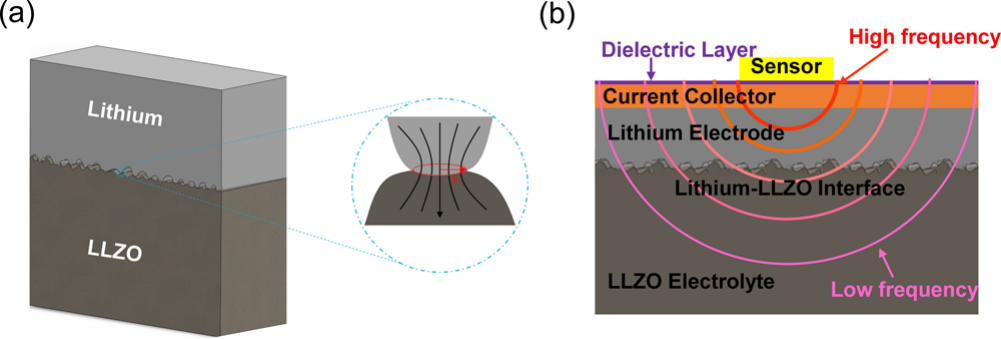
(a) Schematic of the rough lithium–LLZO contact with an expanded view of a single contact. The externally applied pressure leads to lithium deformation at the interface leading to an equilibrium distribution of lithium–LLZO contacts with average radius *a* and number of contacts per unit area *n*. (b) Schematic of the frequency-dependent thermal waves for measuring subsurface thermal properties including the lithium–LLZO thermal contact resistance. The high-frequency waves have shorter penetration depth and probe the properties of layers close to the sensor while the low-frequency waves penetrate deeper up to the electrolyte. The variation in the measurement frequency allows spatially resolved probing of subsurface thermal properties and the isolation of the lithium–LLZO interface resistance.

The 3ω method, based on frequency-dependent thermal penetration depth, , where *D* is the sample’s thermal diffusivity and 2ω is the frequency of the thermal wave, can be used to noninvasively probe the thermal conductivity and thermal resistance of materials and interfaces beneath the 3ω sensor surface (Figure 1b). The spatial resolution in this method is achieved by varying the modulation frequency (ω), which determines the thermal penetration depth (δ_p_) at which the thermal properties are probed. With a 3ω sensor deposited on the current collector, if the thermal conductivity and the volumetric specific heat capacity of the subsurface layers are known, then thermal resistance of the interface of interest (in this case the lithium metal–LLZO interface) can be selectively isolated. The interface resistance thus measured can be related to the interface morphology using an appropriate thermal contact resistance model. Details of the 3ω technique for batteries can be found in the literature.^41,42^

In this work, we use the elastoplastic contact conductance model developed by Yovanovich et al.^43^ to describe the measured thermal interface resistance at the lithium–LLZO interface. We choose the elastoplastic contact conductance model as the pressure studied here is close to the elastic yield strength of lithium, where the deformation mechanics switches from elastic to plastic, and the elastoplastic model is capable of accounting for both deformation mechanisms. The schematic of the interface is shown in Figure 1a. By simplifying the model of Yovanovich,^43^ the measured thermal interface resistance (*R*_int_) can be related to the effective interface conductivity (*k*_int_), pressure (*P*), effective elastoplastic hardness (*H*_ep_), and the surface morphology parameters—absolute surface slope (*m*) and mean surface roughness (σ)—by the relation
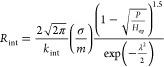
1where
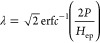
2When the elastic modulus (*E*) ≫ yield strength (*S*_y_), the effective hardness can be approximated as
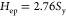
3The absolute surface slope (*m*) and the mean surface roughness (σ) of the two contacting surfaces are related to the surface slopes (*m*_1_ and *m*_2_) and surface roughness (σ_1_ and σ_2_) of the individual contacting surfaces by
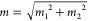
4
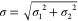
5The effective interface thermal conductivity (*k*_int_) is related to the thermal conductivity of the two contacting surfaces (*k*_1_ and *k*_2_) as
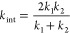
6For both the symmetric and the anode-free cell configurations, we obtain the electrolyte roughness and surface slope (σ_2_ and *m*_2_) from profilometry. To explain the pressure versus interface contact resistance (*R*_int_) relationship in the case of a symmetric cell where the roughness and surface slope of the contacting lithium are not known, we fit an effective lithium roughness parameter (σ_1_) and use a correlation developed by Antonetti et al.^44^ (*m*_1_ = 0.125σ^0.402^) to approximate the surface slope (*m*_1_). For the anode-free cell, we assume that the roughness of the deposited lithium to be that of the electrolyte. We then fit an effective lithium hardness to explain the pressure versus interface contact resistance (*R*_int_) relationship for the LLZO–copper interface.

From the elastoplastic model, it can be shown that even without knowledge of the mean surface slope (*m*) and the effective roughness (σ), if the effective contact hardness (*H*_ep_) and the stack pressure (*P*) are known and the thermal interface resistance (*R*_int_) is measured from the 3ω method, then the mean contact spot size (*a*) and the density of contacts (*n*_contacts_) can be directly extracted as
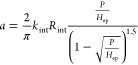
7
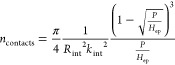
8

## Experimental Methods

### Electrolyte Preparation

Al–LLZO pellets were made from commercially available Al–LLZO powder (500 nm, MSE Supplies) and contained 4 wt % MgO (500 nm, US Research Nanomaterials) to control grain growth and 1 wt % Li_2_CO_3_ to mitigate lithium loss during sintering. The pellets were made by adding the ceramic components, methycellulose (25 cP, Sigma), poly(ethylene glycol) (300, Aldrich), and Dispex Ultra PA4560 (BASF) to water and ethanol in a mass ratio of 55:1:1.2:3.6:70:24. The mixture was ball-milled overnight with ZrO_2_ media, then dried, crushed by a mortar and pestle, and pelletized with a 3/4 in. die at 160 MPa pressure. Prior to sintering, the green pellets were debinded by heat treatment in air at 675 °C for 4 h.

**Figure 2:**
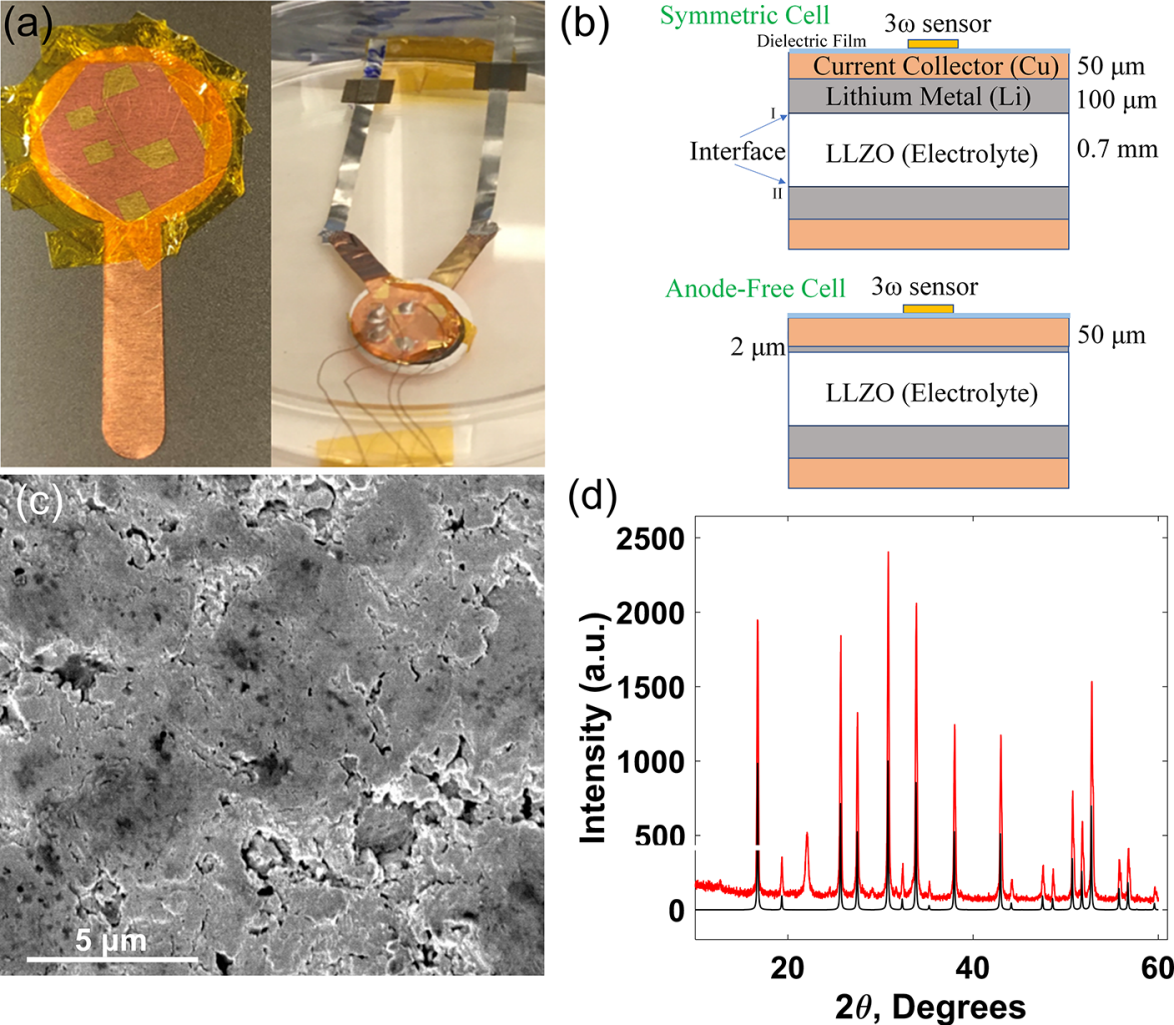
(a) A 3ω sensor deposited on a copper current collector (left) and assembled in a symmetric configuration (right). (b) Schematic of the symmetric (top) and anode-free (bottom) configuration. (c) SEM image of the LLZO pellet surface showing fused grains. (d) XRD pattern of the LLZO pellet (red) compared with the reference (black, generated from the CIF on Crystallography Open Database^45^).

Pellets were sintered using pyrolytic graphitic carbon sheets (Panasonic) as a substrate under flowing argon in a tube furnace. The ramp rate was 5 °C/min to 700 °C and 2 °C/min to 1050 °C. Sintered pellets were approximately 90% dense. The pellet surface under SEM is shown in Figure 2c, and XRD of the pellet is shown in Figure 2d.

### Sensor Fabrication and Cell Assembly

A dielectric film with a laminate structure of 200 nm alumina (e-beam evaporation), 500 nm parylene C (chemical vapor deposition), and 200 nm alumina (e-beam evaporation) was deposited on a 50 μm copper current collector. A 4-point 3ω sensor (Figure 2a) was deposited on the dielectric layer by e-beam evaporation of 100 nm of gold with a 10 nm chromium adhesion layer. For the cell assembly, alumina–LLZO pellets were polished and annealed in a tube furnace with argon at 700 °C for 4 h to remove the surface contaminants, and 50 nm of gold (e-beam evaporation) was coated on both sides of the annealed pellets. In the next step, 12 mm diameter discs of 100 μm thick lithium foil (MSE Supplies) lithium were pressed onto the LLZO pellet either on both sides (symmetric cell) or one side (“anode-free” cell) of the LLZO pellet, shown in Figure 2b. The structure was then sandwiched between two copper current collectors connected to nickel tabs, with the fabricated 3ω sensor on one current collector. The sandwich structure was heated to ∼200 °C to melt the lithium and bond with the LLZO pellet. A 2–3 mm styrofoam was attached on top of the 3ω sensor to provide thermal insulation,^41,42^ and the cell was finally sealed in a pouch cell configuration. After assembly, a 2 μm lithium film was deposited on the sensor side of the anode free cell by passing 450 μAh equivalent lithium from the counter electrode (nonsensor side). The process of sensor fabrication and cell assembly is described in detail in the Supporting Information.

### Thermal Interface Resistance Measurement

The thermal interface resistance at the lithium–LLZO interface was measured by the 3ω method based on bidirectional multilayer heat flow analysis^46^ using Feldman’s algorithm.^47^ The detailed thermal analysis is presented in our previous works,^41,42^ and a representative fitting as well as the thermal properties of each layer involved is presented in the Supporting Information. The uncertainty in the measurements is calculated from uncertainties of parameters used in the data fitting (see Table S1). For the 3ω measurements, the temperature coefficient of resistance (TCR) of each sensor was measured by 4-point resistance measurement at temperatures in the range 25 to 40 °C (see Figure S2). AC current through the sensor was provided by a Keithley 6221 current source, and the subsequent 3ω voltage was measured with an SR830 lock-in amplifier.

### Electrochemical Tests

The electrochemical tests including galvanostatic cycling and EIS measurements were done using a Biologic VMP3 multichannel potentiostat. Galvanostatic cycling was performed at 20 μA current (17.68 μA/cm^2^) with a voltage limitation of +–5 V to pass 450 μAh lithium (equivalent to ∼2 μm) between the two sides of LLZO. Potentiostatic EIS measurements were performed between 1 MHz and 1 Hz with 50 mV amplitude and no DC offset.

### Ex-Situ Characterization

Roughness measurements were done via optical profilometry with a Keyence VK-X1000 3D surface profiler using laser confocal microscopy at 20× magnification. The lateral resolution for the measurement was 220 nm (diffraction limit), and the height resolution was 5 nm. SEM measurements were done using an FEI Quanta 3D FEG dual beam electron microscope (UC Berkeley Biomolecular Nanotechnology Center Cleanroom). For the characterization of samples with lithium, pouch cells were cut open, and the LLZO pellets were quickly transferred to the SEM chamber to minimize the exposure to air.

## Results and Discussion

### Measurement Sensitivity and Thermal Characterization of LLZO

**Figure 3:**
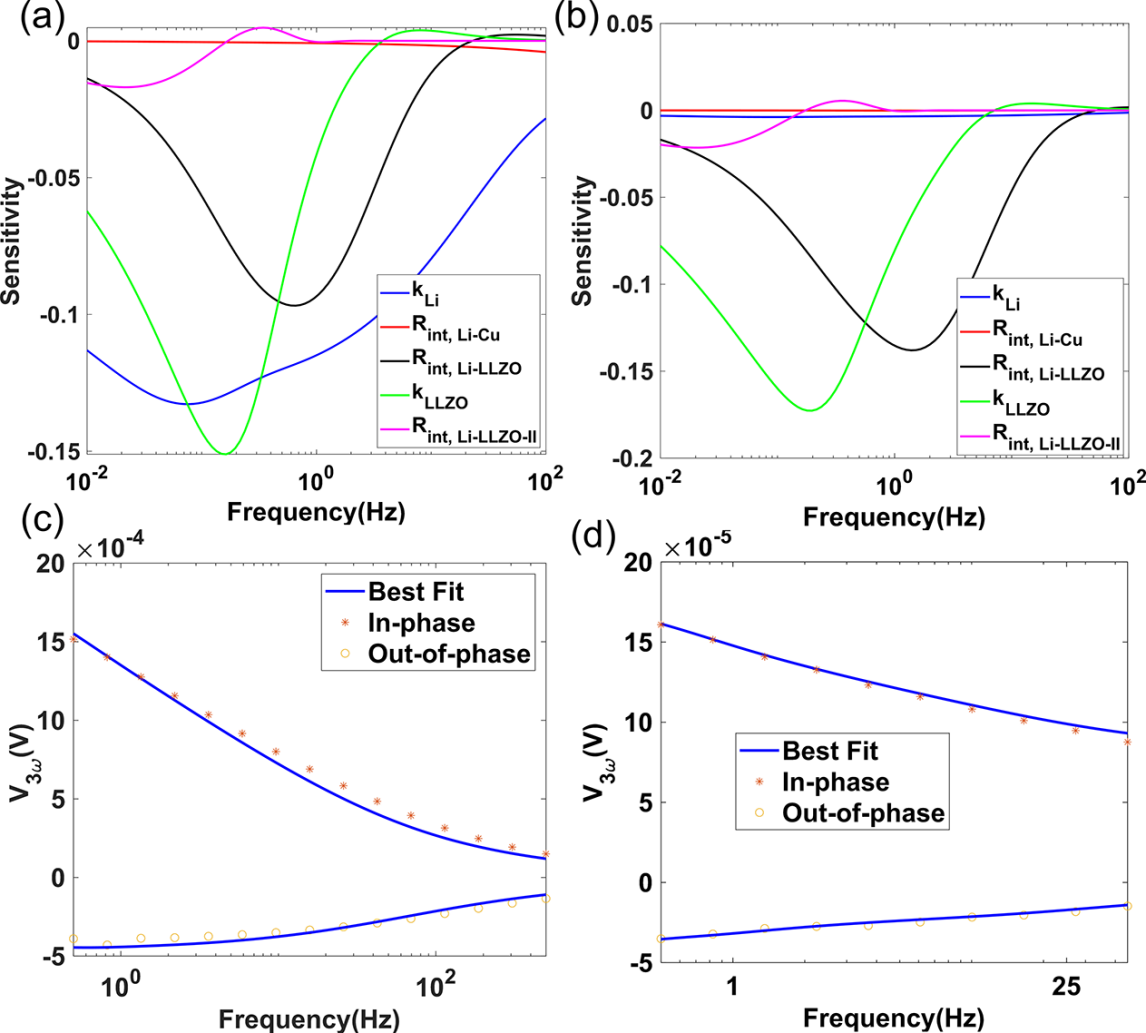
Absolute measurement sensitivity for thermal properties of different layers as a function of the measurement frequency for (a) symmetric and (b) anode-free cells, (c) 3ω measurement of LLZO thermal conductivity with the 3ω sensor deposited on a LLZO pellet, and (d) a representative 3ω measurement of a symmetric lithium–LLZO cell. From the best fit shown, the thermal interface resistance for the symmetric cell at 750 kPa external pressure was obtained to be 2.7 × 10^5^ m^2^ K/W.

In thermal wave sensing based on the 3ω method, the absolute measurement sensitivity for a particular parameter *p* is defined as , where *V*_3ω_ is the magnitude of the 3ω voltage measured. The sensitivity analysis (Figure 3a,b) reveals that the 3ω voltage is the most sensitive to the lithium–LLZO interface between 0.1 and 1 Hz 1ω (AC current) frequency for both symmetric and anode-free cells. The absolute measurement sensitivity to the interface is higher in the case of the anode-free cell because of the interface being close to the sensor. To optimize the measurement sensitivity, we perform 3ω measurements from 45 to 0.5 Hz as shown in Figure 3d. To measure the thermal interface resistance, we fit the bidirectional multilayer 3ω model^46^ to the measured 3ω voltage (*V*_3ω_) and extract the effective thermal conductivity of the alumina–parylene–alumina dielectric layer at shorter thermal penetration depths (high frequency, ∼30 to 45 Hz) and subsequently the effective Li metal–LLZO thermal interface resistance at longer thermal penetration depths (low frequency, 0.5 to 10 Hz). The 3ω voltage, particularly at low frequencies, is sensitive to the thermal conductivity (*k*) and the volumetric specific heat capacity (*C*_*p*_ = density (ρ) × mass specific heat (*c*_*p*_)) of LLZO. The average specific heat capacity (*c*_*p*_) was obtained to be 618 J/(kg K) from differential scanning calorimetry (DSC). The density was measured to be 3.894 g/cm^3^, and the thermal conductivity was determined to be 1.33 W/mK from the 3ω method (see Figure 3c).

### Measurement of Lithium–LLZO Surface Morphology

#### Interface Evolution with Pressure

**Figure 4:**
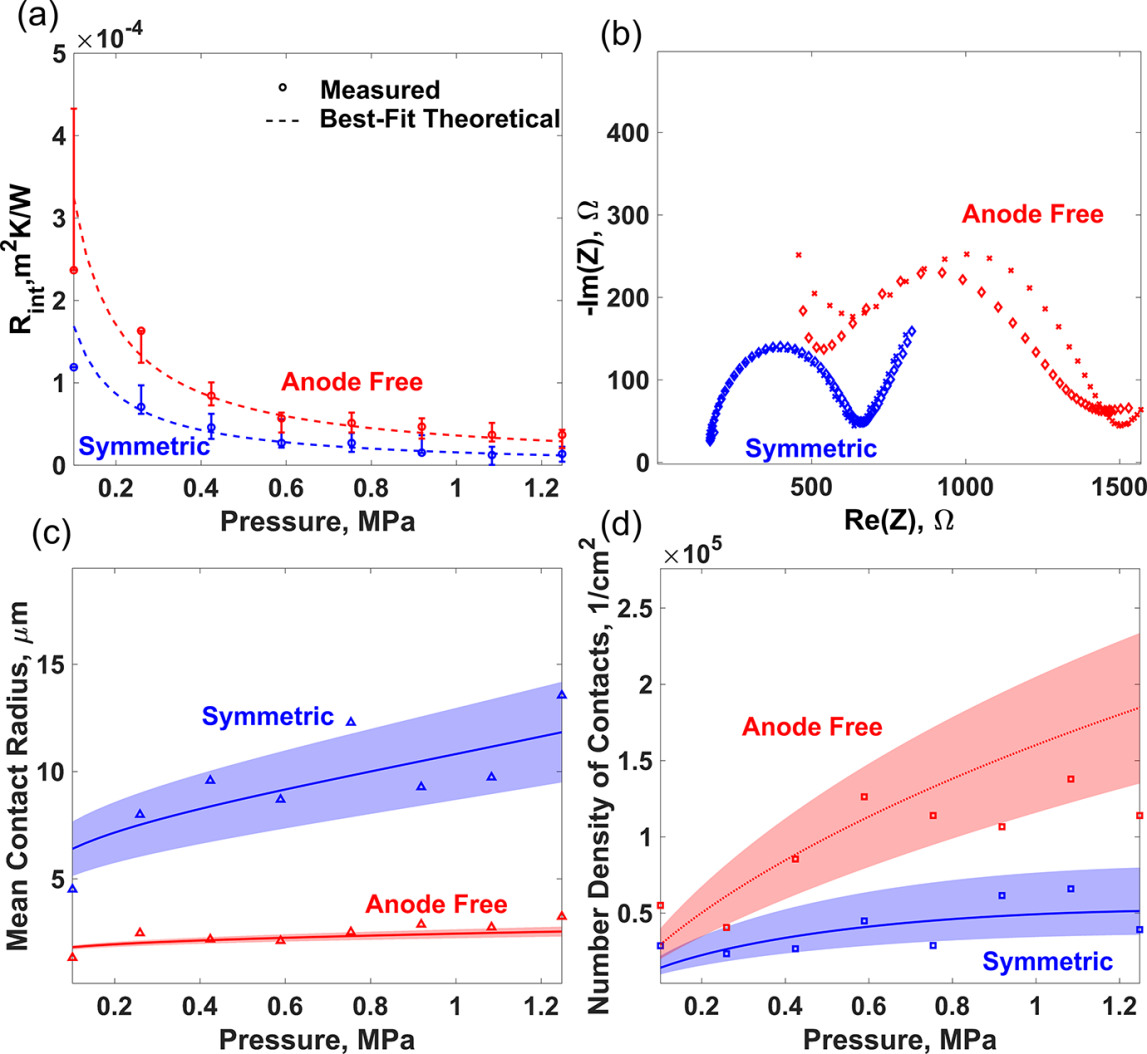
(a) Measured thermal interface resistance as a function of external stack pressure for anode-free (red) and symmetric (blue) cells. The theoretical best-fit lines (dashed) are obtained by fitting the interface roughness (σ) for the symmetric cell and the effective hardness for the anode-free cell. (b) EIS Nyquist plots for the symmetric (blue) and the anode-free (red) cells at atmospheric pressure (diamonds) and 1.25 MPa (crosses). There is no significant dependence of EIS spectra with pressure as the interface behavior is dominated by gold deposited on the electrolyte. Calculated mean contact radius (c) and number density of contacts (d) as a function of pressure for the symmetric (blue) and the anode-free (red) cells. The shaded areas show the error bands in the theoretical estimates from the 3ω measurements.

We performed simultaneous 3ω measurements and electrochemical impedance spectroscopy (EIS) measurements on freshly assembled (uncycled) symmetric cells and anode-free cell with 2 μm lithium plated on the anode to extract the thermal interface resistance and the electrochemical interface impedance, respectively, as a function of pressure from atmospheric (101 kPa) to 1.2 MPa pressure using a custom setup with a calibrated pressure gauge (see the Supporting Information). Figure 4b shows the EIS Nyquist plots at atmospheric and 1.2 MPa pressures for the symmetric cell and the anode free cell. See the Supporting Information for the Nyquist plots at intermediate pressures. As seen from the figure, we did not observe a strong pressure dependence of the electrochemical impedance for both symmetric and anode free cells. We hypothesize that this is caused by the fact that in both the symmetric and the anode free cell the electrochemical interface behavior at the LLZO–lithium interface is dominated by the thin gold–lithium layer that forms when lithium melts onto the gold coated on LLZO. The gold–LLZO contact does not change significantly with pressure, and therefore the electrochemical interface resistance does not change with pressure. This is corroborated by the tail seen in the EIS plots which is characteristic of the ion-blocking gold electrode.^48,49^ To validate this observation further, we performed electrochemical simulations of the overpotential at the lithium–LLZO interface as a function of pressure for a rough lithium–LLZO contact with and without the presence of a thin gold layer. As expected, we observed that the interface overpotential and hence the interface impedance are in fact unaffected by external pressure in the presence of a thin gold layer while the interface overpotential varies with pressure when there is no gold layer present. Please refer to Figures S8 and S9 and the accompanying discussion in the Supporting Information for additional details regarding the electrochemical simulation of the interface.

Unlike the electrochemical impedance, the thermal interface resistance, which is dominated by the morphology of the interface (eq 1), varies strongly with pressure, and we observe a pressure dependence (Figure 4a) expected from the elastoplastic contact conductance models.^43,50^ In the case of the symmetric cell (Figure 2b), with bulk lithium (100 μm) between LLZO and the current collector, we assume that the lithium hardness at the interface remains the same as that of the bulk lithium and fit the roughness parameter (σ_1_) to explain the pressure–thermal interface resistance behavior. The best fit is obtained for lithium roughness of 2.5 μm. However, in the case of the anode-free cell, where a thin film of lithium (∼2 μm) is between the LLZO and the current collector, the lithium hardness is expected to be greater than that of the bulk.^51^ Therefore, to explain the pressure vs thermal interface resistance data, we assume the lithium roughness to be the same as that of LLZO (uniform deposition) and vary the lithium yield strength in the elastoplastic thermal conductance model to obtain the best fit. The best fit was obtained for a lithium yield strength of 12 MPa. This value is within the range of the yield strength reported in the literature^51^ for a 2 μm lithium film and further validates the applicability of the elastoplastic thermal conductance model. Once the effective hardness (or yield strength) is known, the measured thermal interface resistance can directly be used to extract the average morphological information on the interface, namely, the mean contact radius and the number density of contacts using eqs 7 and 8, respectively. Figures 4c and 4d respectively show the evolution of the effective contact radius and the number density of contacts with pressure for both the anode-free and the symmetric cells. As expected, both the contact spot size and the contact density increase with pressure as new contacts are formed and existing contacts become bigger with the increase in pressure. Also, the pressure dependence of both the mean spot size and the number density is stronger in the case of the symmetric cell because of lower effective lithium hardness leading to easier deformation.

#### Interface Evolution with Cell Cycling

**Figure 5:**
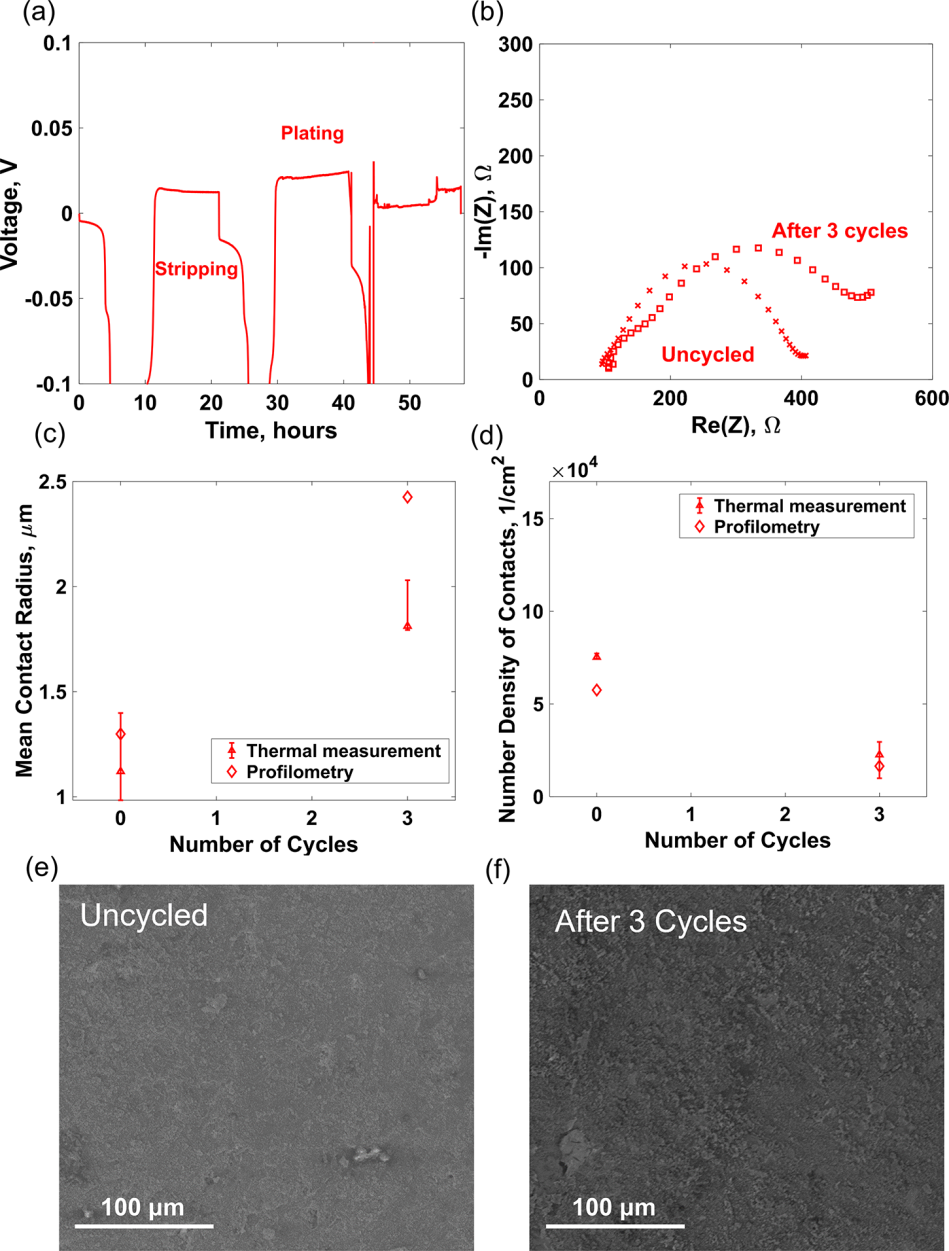
(a) Voltage vs time plot for the galvanostatic cycling with potential limitation (GCPL) of the anode-free cell. Due to lithium depletion during stripping, a large overpotential is developed and the cut-off potential (5V) is reached. The overpotential associated with the plating process is small but increases steadily with the number of cycles. This increase in impedance is also observed in the EIS spectra (b) and can be attributed to void formation and migration of the lithium-gold layer away from the LLZO surface. Mean contact radius (c) and number density of contacts (d) measured from thermal interface resistance (triangles) and profilometry (diamond) on anode-free cells. The results from thermal-contact resistance measurement agrees well with the results from profilometry and capture a general trend of interface degradation (decrease in contact density and increase in individual contact size) which is further verified by SEM images of lithium deposited on the LLZO surface (e and f).

Because the measured thermal interface resistance can be related directly to the interface morphology from eqs 7 and 8, we cycled both symmetric and anode-free cells and performed simultaneous 3ω measurements to observe the interface morphology evolution with cycling. The cell cycling and the 3ω measurements were done at atmospheric pressure, i.e., without applying any external pressure. In the case of anode-free cells, we could also perform ex-situ measurements of the interface profile, through optical profilometry and SEM, which provides a direct method of comparing and verifying the measurements from the 3ω method. Therefore, the results presented here are only for the anode free cells. The interface profile for the symmetric cells obtained from the 3ω method is presented in the Supporting Information. Figure 5a shows the voltage vs time plot for the galvanostatic cycling with potential limitation (GCPL) of the anode-free cell. By considering the “anode-free” side as the reference electrode, we can define the movement of lithium toward the anode-free side as plating and away from the anode free side as stripping. As observed, the overpotentials associated with the plating and the stripping process are not symmetric. During stripping, the 2 μm lithium that was initially plated onto the electrode is moved away toward the counter electrode. Because of lithium depletion in the anode-free side, a large polarization develops, and the cutoff voltage of −5 V is reached. During plating, however, because of virtually unlimited lithium supply in the counter electrode, such polarization is not observed, and the overpotential associated with the plating process is small. This overpotential, however, increases gradually with the number of cycles and can be associated with the formation of interfacial voids and a possible migration of the gold–lithium layer away from the electrolyte surface. This behavior is corroborated by the increase in the impedance measured after the three cycles compared to the uncycled cell (Figure 5b). From the 3ω measurements, the measured mean spot radius and number density for the anode-free cells are shown in Figures 5c and 5d, respectively. As seen, we observed that the thermal interface resistance increases with cycling, leading to decreased number density of contacts (red triangles in Figure 5d). Because of the constant external pressure leading to plastic deformation, as the number density of contacts decreases, individual contacts become bigger to maintain a force balance at the interface, which is indicated by the increase in the average contact radius after three cycles as shown in Figure 5c. We performed optical profilometry measurements of the electrolyte pellet preassembly and the deposited lithium after three cycles and calculated the mean contact radius and the contact number density from the measured profile. As shown by the red diamonds in Figures 5c and 5d, the measured values were close to what was obtained from the thermal measurements, and the qualitative trend of the increase in the interface roughness with cycling was confirmed. We assembled two additional anode-free cells with similar electrolyte roughness and performed SEM imaging on one of the cells after the initial 2 μm lithium plating (uncycled, Figure 5e) and on another cell after three cycles (Figure 5f). As seen, the SEM images also confirm the increase in roughness with cycling, which further corroborate the qualitative trend observed from the thermal interface resistance measurements.

## Limitations and Outlook

The measurements performed in this work were limited by the sensor durability at high pressures. We were only able to perform our experiments at a maximum pressure of 1.25 MPa, mainly because at higher pressures, the silver epoxy used to bond wires on the sensor pads (see Figure 2a) punctured the dielectric insulation film (Figure 1b), causing the sensor to short with the current collector. In the future, this problem can be mitigated by changing the sensor design to make wire connections away from the stack on which the pressure is applied. Additionally, the main source of uncertainty (error bars) in the results presented here comes from the uncertainty in lithium metal thermal conductivity (estimated to be 5% of the standard value, see Table S1). The overall measurement uncertainty can be improved if a lithium foil thinner than 100 μm is used.

In addition, the elastoplastic thermal contact conductance model used in this work assumes nominally planar rough surfaces in contact. In an actual solid-state battery, the electrode–electrolyte architecture might be more complex in the presence of specially designed or porous electrodes,^52,53^ in which case the elastoplastic contact conductance model cannot be directly applied. However, the measured thermal interface resistance can still be related to the electrode–electrolyte contact through more complex thermomechanical modeling using finite-element or other numerical methods. Additionally, unlike in the presence of voids, which significantly increases the interface thermal contact resistance, the propagation of dendrites into the solid electrolyte does not change the thermal contact resistance of the interface significantly. While the presence of a metallic lithium in the low thermal conductivity ceramic (LLZO) might increase the interface thermal conduction slightly, we assume that this effect is not observable in the 3ω measurement. Therefore, the presented method cannot directly be used to observe interfacial dendrite growth. Finally, the elastoplastic contact conductance model used here is valid only when the applied nominal pressure is less than the lithium hardness. In cases where the applied pressure is comparable to or greater than the lithium hardness, the contact mechanics is dominated by creep behavior,^20^ which needs to considered while modeling the thermal contact resistance.

## Conclusions

Operando monitoring of buried interfaces in solid-state battery cells has proven to be difficult with traditional methods that either modify the interface or require complicated experimental setups and analyses. In this work, we present a simple method of operando observation of the lithium–solid-state electrolyte interface morphology from measurement of the thermal interface resistance enabled by thermal wave sensing. Morphological parameters such as the mean contact radius and the number density of contacts have been extracted from thermal measurements by considering the effect of morphology and contact mechanics on the solid–solid thermal interface resistance. By utilizing the frequency dependence of thermal penetration depth, the method provides spatial resolution to attribute the observed interface resistance to specific interfaces which is an ability not possible with measurement techniques such as EIS. Although the results presented in this work relate to nominally planar rough surfaces in contact with each other at low to moderately high stack pressures (0.1 to 1.25 MPa), this method can be applied to more complex electrode architectures at higher pressures by modifying the sensor design and extending the thermal contact model.

## Supporting Information Available

Details of the sensor fabrication, details of the cell assembly procedure, specific heat capacity of LLZO, temperature coefficient of resistance measurement, representative 3ω fitting for anode-free cell, external pressure application and measurement setup, EIS Nyquist plots, symmetric cell cycling measurements, non-gold-coated cell measurement, interface overpotential simulations, thermomechanical model comparison, thermophysical properties, and uncertainties used in the 3ω model (PDF)
