# Supplementary material for: Using Thermal Interface Resistance for Noninvasive Operando Mapping of Buried Interfacial Lithium Morphology in Solid-State Batteries
Source: ACS Appl Mater Interfaces. 2023 Mar 23;15(13):17344–52. doi: 10.1021/acsami.2c23038 (PMC10080538; doi:10.1021/acsami.2c23038)
Supplement: Supplementary file 1 — am2c23038_si_001.pdf [file am2c23038_si_001.pdf]

Supporting Information

Using thermal interface resistance for non-invasive operando mapping of buried interfacial lithium morphology in solid-state batteries

Divya Chalise[1,2], Robert Jonson[2], Joseph Schaadt[1], Pallab Barai[3], Yuqiang Zeng[2], Sumanjeet Kaur[2], Sean Lubner[2,4], Venkat Srinivasan[3], Michael Tucker[2], Ravi Prasher[1,2,*]

[1] – Department of Mechanical Engineering, University of California, Berkeley, California, 94720, USA
[2] – Energy Technologies Area, Lawrence Berkeley National Lab, 1 Cyclotron Road, Berkeley, California 94720, USA
[3] – Argonne National Laboratory, Lemont, Illinois, 60439, USA
[4] – Department of Mechanical Engineering, Boston University, Boston, Massachusetts, 02215, USA
[*] – Corresponding Author: Ravi Prasher: rsprasher@lbl.gov

Contents:

Details of the Sensor Fabrication

Details of the Cell Assembly Procedure

Figure S1. Specific heat capacity of LLZO as a function of temperature obtained from Differential Scanning Calorimetry (DSC) measurement.

Figure S2. Representative temperature coefficient of resistance measurement for a 3ω sensor.

Figure S3. Representative 3ω fitting to extract the interface resistance.

Figure S4. Setup for application and measurement of external pressure on a pouch cell.

Figure S5. Electrochemical Impedance Spectroscopy (EIS) Nyquist Plots at different pressure for (a) symmetric and (b) anode free cell

Figure S6. (a) Average contact radius and (b) number density of contacts for a symmetric cell as a function of number of cycles.

Figure S7. Measured thermal interface resistance as a function of pressure for a symmetric cell assembled without melting the lithium on gold coated electrolyte.

Table S1. Thermophysical properties and uncertainties used in the 3ω model

Interface Overpotential Simulations

Figure S8. (a) Schematic of the mesh used to simulate the overpotential at the lithium-LLZO interface, (b) overpotential plotted as a function of the applied external pressure and an inset (c) showing stress-potential coupling induced overpotential increase.

Figure S9. (a) Schematic of the mesh used to simulate the overpotential at the lithium-LLZO interface in the presence of a gold layer, (b) zoomed section of the mesh and (c) overpotential plotted as a function of the applied external pressure.

Figure S10. Contact area fraction for the lithium-LLZO contact calculated from the single-contact model and Yovanovich's thermo-mechanical model[9].

Table S2. List of governing equations (GE) used in the electrochemical computational analysis and relevant boundary conditions (BCs).

Table S3. List of parameters used for the interface overpotential simulations

Details of the Sensor Fabrication Process

50um copper sheets (McMaster-Carr) were cut into 12mm diameter circles with protruding ends (see Figure 2(a)) to attach Nickel-tabs for making current collectors. A dielectric film consisting of 200nm alumina, 500nm parylene C and 200nm alumina respectively was deposited on one side of the current collector. Alumina was deposited via e-beam evaporation of aluminum-oxide, and Parylene was deposited via Chemical Vapor Deposition. While parylene worked as the functional part of the dielectric film to electrically insulate the top of the copper sheet, the 200nm alumina layer between the copper sheet and the parylene film improved the adhesion between parylene and copper. The top alumina film was deposited to improve the adhesion between the dielectric film and a metallic 3ω sensor which was deposited on top. The 3ω sensor with a metallic line (150µm wide and 3mm long, Figure 2(a)) and 4 attachment pads, 2 each for passing current and measuring the voltage, was deposited via subsequent e-beam evaporation of 10nm chromium and 100nm gold through a laser-cut shadow mask. Electrical connections were made to the sensor pads by attaching 50µm diameter insulated copper wires using silver epoxy (EPO-TEK® H20E).

Details of the Cell Assembly Procedure (Symmetric and Anode-free)

LLZO pellets were polished on both sides with 15µm alumina lapping films (Ted Pella) and annealed in a tube furnace with Argon at 700°C for 4 hours to remove the surface contaminants. 20nm gold was coated on both sides of the annealed pellets and the inner surface of the copper current collectors via e-beam evaporation to promote lithium wettability on the LLZO surface and copper[1]. 12mm diameter discs of 100um thick lithium foil (MSE) were punched and cleaned on both sides with a tweezer to remove the surface contaminants. The cleaned lithium was then pressed onto the LLZO pellet either on both sides (symmetric cell) or one side ('anode-free' cell) of the LLZO pellet, shown in Figure 2(b). The structure was then sandwiched between two copper current collector attached to nickel tabs. A prefabricated 3ω sensor on the dielectric film was on the outer surface of one of the current collectors (on the side without lithium in the case of anode-free cells). The sandwiched structure was heated to ~200°C at which the lithium melted and bonded with both the copper and the LLZO pellet. After melting, heating was turned off and the assembly was allowed to cool to room temperature. A 2-3mm thick Styrofoam was attached on top of the 3ω sensor to work as a thermal insulation[2,3] and the cell was finally sealed in a pouch cell configuration, the process of which is described in our previous work[3].

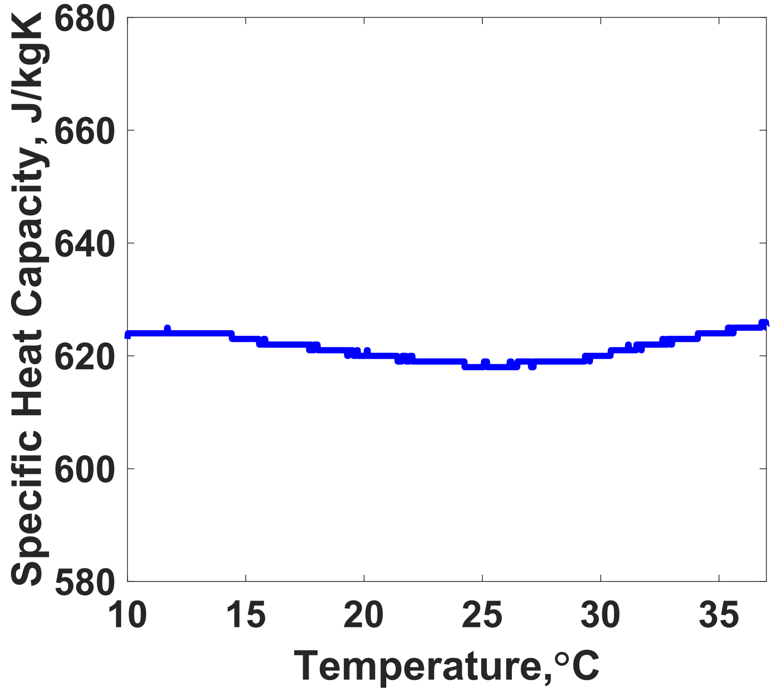

Figure S1. Specific heat capacity of LLZO as a function of temperature obtained from Differential Scanning Calorimetry (DSC) measurement.

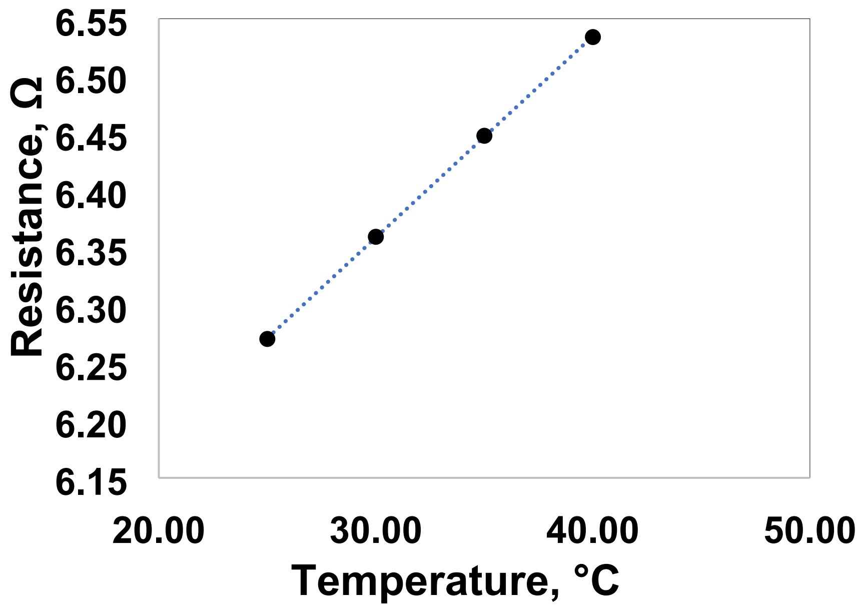

Figure S2. Representative temperature coefficient of resistance measurement for a 3ω sensor from resistance measurement at four different temperatures. From a linear fitting, the obtained values of resistance (R) at 25°C is 6.272Ω and the temperature coefficient of resistance (dR/dT) is 0.0175Ω/K.

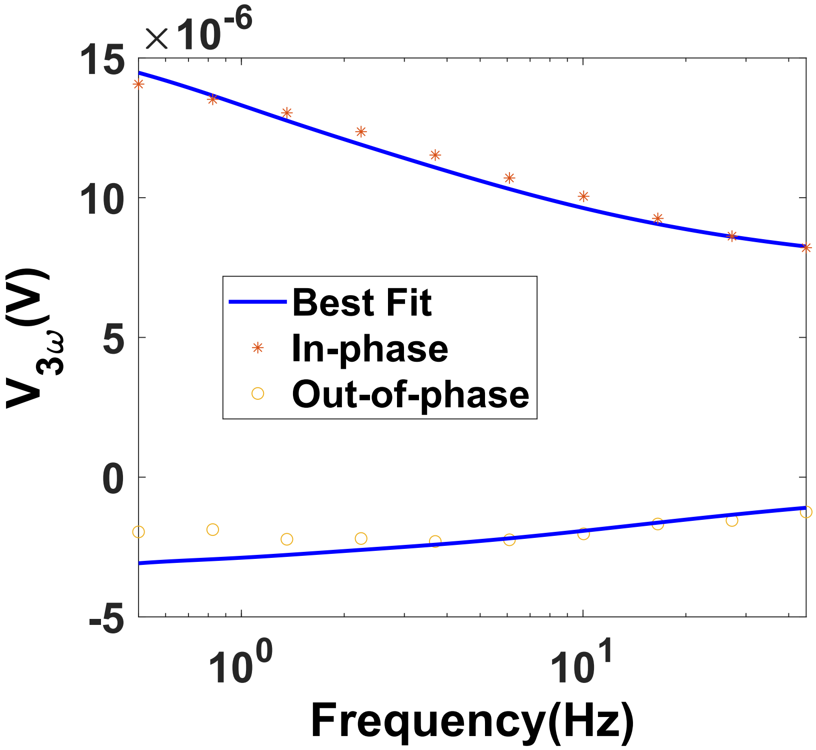

Figure S3. Representative 3ω fitting to extract the interface resistance for an anode-free cell. From the best fit shown above, the thermal interface resistance for the cell at 425kPa external pressure was obtained to be $8.43X10^{-5}\ m^2K/W$.

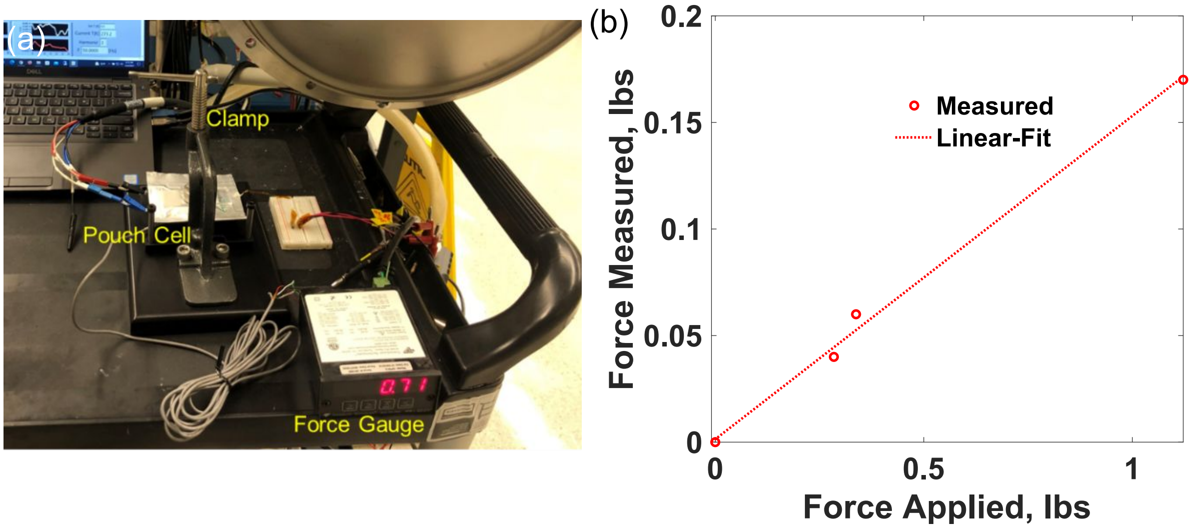

Figure S4. (a) Setup for application and measurement of external pressure on a pouch cell with a clamp and a force gauge system and (b) calibration of the force gauge with a linear fitting to obtain a calibration factor of 6.5933. The setup was designed by Kenneth Higa (LBL).

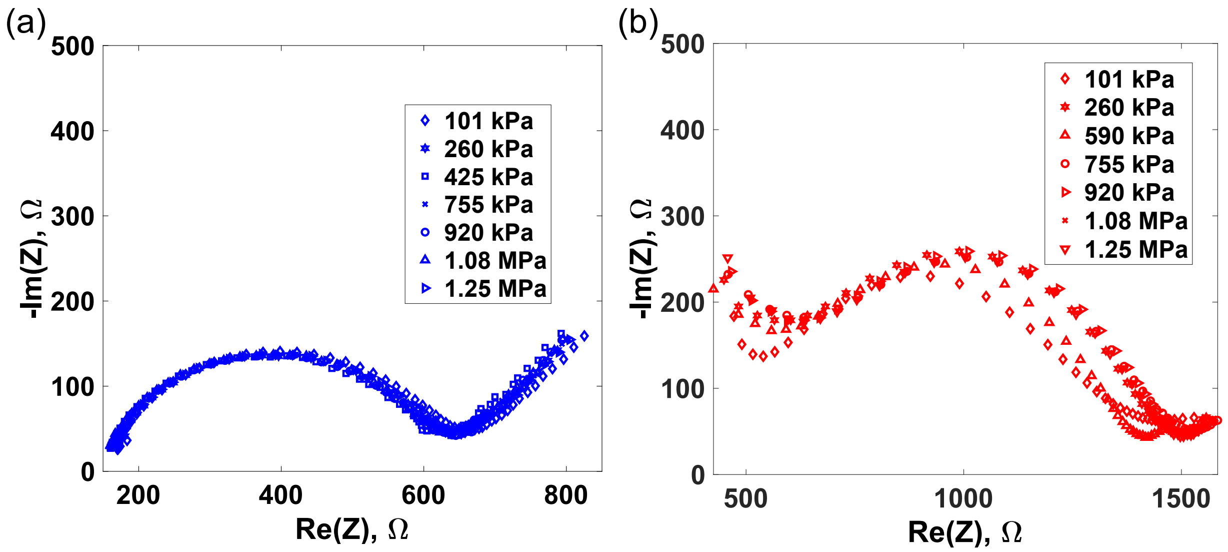

Figure S5. Electrochemical Impedance Spectroscopy (EIS) Nyquist Plots at different pressure for (a) symmetric and (b) anode free cell

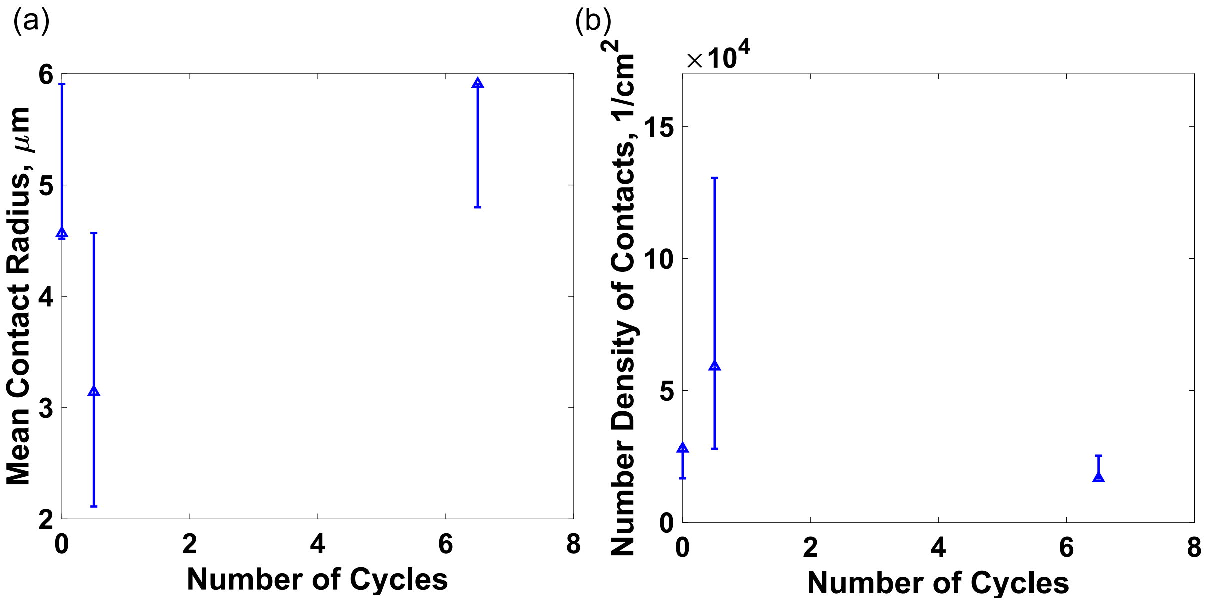

Figure S6. (a) Average contact radius and (b) number density of contacts for a symmetric cell as a function of number of cycles. The interface resistance first decreases with the initial plating (0.5 cycles), which we hypothesize is because of plating filling the voids that were formed during the assembly. Upon further cycling, the thermal interface resistance increases. This translates into

increase in the number density of contacts and a decrease in the average contact radius after the first plating and a decrease in contact density and an increase in the average contact radius after 6 cycles.

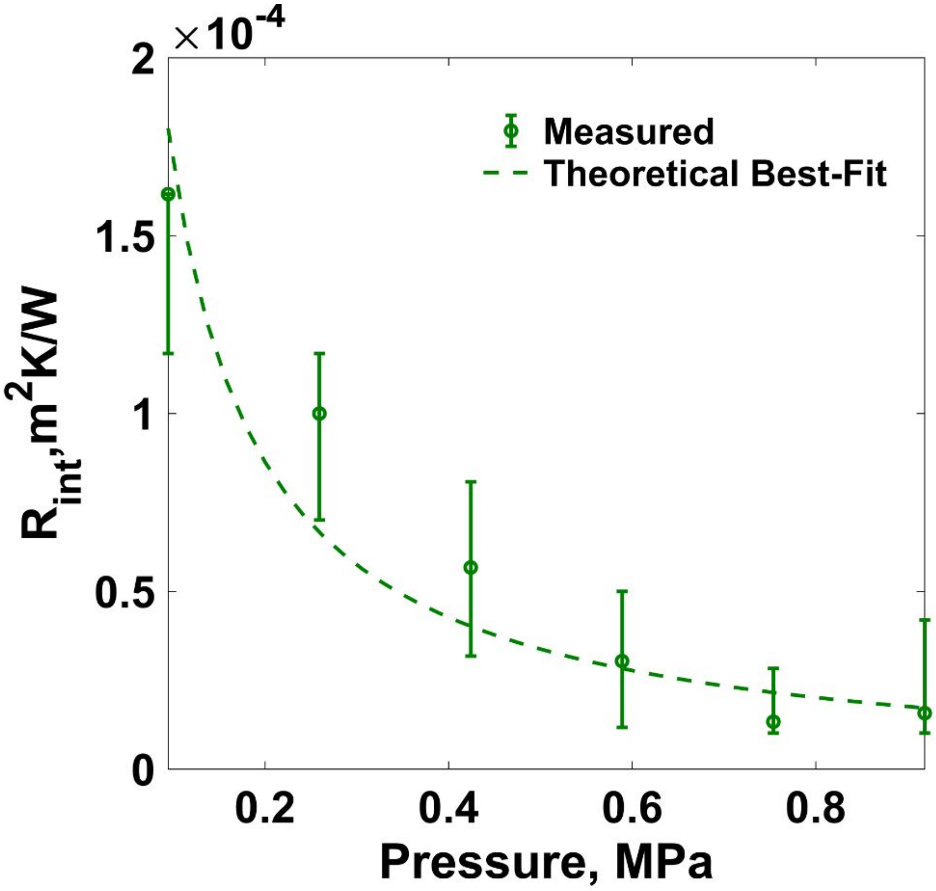

Figure S7. Measured thermal interface resistance as a function of pressure for a symmetric cell assembled without melting the lithium on gold coated electrolyte. The magnitude and the pressure dependence of the interface resistance is similar to that of the symmetric cell presented in Figure 3 (a) indicating that the thermal interface resistance is largely independent of the presence of a thin gold-lithium alloy at the interface.

Table S1. Thermophysical properties and uncertainties used in the 3ω model

Material	k (W/m-K)	$\Delta k/k$	C (kJ/m^{3}K)	$\Delta C/C$	L (µm)	$\Delta L/L$
**Alumina**[2]	1.7	5.9%	3080	6.5%	0.2	5.0%
**Parylene**[4]	Based on fit	Based on fit	960	5.0%	0.4 to 0.6	5.0%
**Cu foil**[5]	401	5.0%	3440	5.0%	55*	2.0%*
**Cu-lithium**	Based on fit	Based on fit	10^{-3}	-	10^{-3}	-
**Lithium Foil**[6]	85	5%	1913	%	100*	2%*
**Lithium-LLZO**[*]	Based on fit	Based on fit	10^{-3}	-	10^{-3}	-

**LLZO***	1.33	5.0%	2407	5.0%	700*	10%**
**Styrofoam**[3]	0.024	10%	16	20%	3000*	5.0%**

*Measured in-house, **estimated

Interface Overpotential Simulations

To investigate the impact of external pressure on the interface between Li electrode and LLZO solid electrolyte, an electrode/electrolyte mesh is generated as depicted in Figure S9(a) where the Li electrode exists at the bottom and the LLZO electrolyte is located at the top. To recreate the interfacial imperfection between the Li and LLZO, a sinusoidal oscillation is provided to the LLZO solid electrolyte, while only the left most node is kept in contact under zero external pressure. Wavelength and amplitude of the sinusoidal shape is extracted from the experimentally predicted roughness of the Li/LLZO interface. Pressure is applied from the top in an incremental fashion, and the deformation of the Li and LLZO domains is estimated by solving force equilibrium relations. Both elastic and plastic deformation of lithium is taken into consideration, whereas only elastic deformation of LLZO is assumed in the developed computational methodology. As pressure is applied and the LLZO nodes reach close enough to the Li nodes, they are assumed to touch each other and remain in contact during the application of the rest of the pressure.

Electrochemical response of the combined electrode/electrolyte system is determined by solving charge balance equations within both the LLZO solid electrolyte and Li metal electrodes. LLZO is assumed to be a single ion conductor (SIC), which carries ions through only the migration process. No grain/grain-boundary microstructure of the LLZO electrolyte is taken into consideration in the developed model. Charge transport within Li metal electrode occurs through the migration of electrons. Potential distribution within both Li and LLZO is captured by solving appropriate Laplace equations that considers the correct magnitude of the conductivity of that phase. Lithium ions carried by the LLZO solid electrolyte electrochemically react with the electrons from the Li electrode side and deposit at the electrode/electrolyte interface as Li metal. The reaction current at the Li/LLZO interface (i_{BV}) is given by the nonlinear Butler-Volmer equation, which is written as[7,8]:

$$i_{\mathrm{BV}} = i_0 \exp\left(\Delta\mu_{\mathrm{e}^-}/2RT\right) \cdot \left[\exp\left(F\eta_{\mathrm{s}}/2RT\right) - \exp\left(-F\eta_{\mathrm{s}}/2RT\right)\right] \qquad \text{(S1)}$$

where, i_0 indicates the exchange current density, F is the Faraday constant, R indicates the universal gas constant, T is temperature, $\Delta\mu_{\mathrm{e}^-}$ is the stress induced electrochemical potential term and η_{s} is the surface overpotential that is defined as, $\eta_{\mathrm{s}} = \phi_{\mathrm{s}} - \phi_{\mathrm{e}} - U_{\mathrm{Li}} + \left(\Delta\mu_{\mathrm{e}^-}/F\right)$, where ϕ_{s} is the potential in Li electrode, ϕ_{e} is the potential in the electrolyte, and U_{Li} indicates the open circuit potential for lithium deposition (in general, $U_{\mathrm{Li}} \sim 0.0\,V$). Magnitude of the stress induced electrochemical potential $\left(\Delta\mu_{\mathrm{e}^-} \sim \bar{V}_{\mathrm{Li}} p_{\mathrm{Li}}\right)$ depends on the mechanical stress state of the Li metal electrode (p_{Li}) and molar volume of lithium $(\bar{V}_{\mathrm{Li}})$. Molar volume of Li ions in the solid electrolyte is assumed to be zero in this analysis, which is consistent with the single ion conducting behavior of the LLZO solid electrolytes. With increasing pressure, the contact between the Li electrode and LLZO solid electrolyte gets better, and the potential drop associated with charge transfer between the electrode and electrolyte should decrease. Note that in the computational simulations, zero potential boundary condition is applied on top of the LLZO electrolyte, and constant current boundary condition is applied at the bottom of the Li electrode. Zero current is assumed on the left and right sides of the computational domain.

.

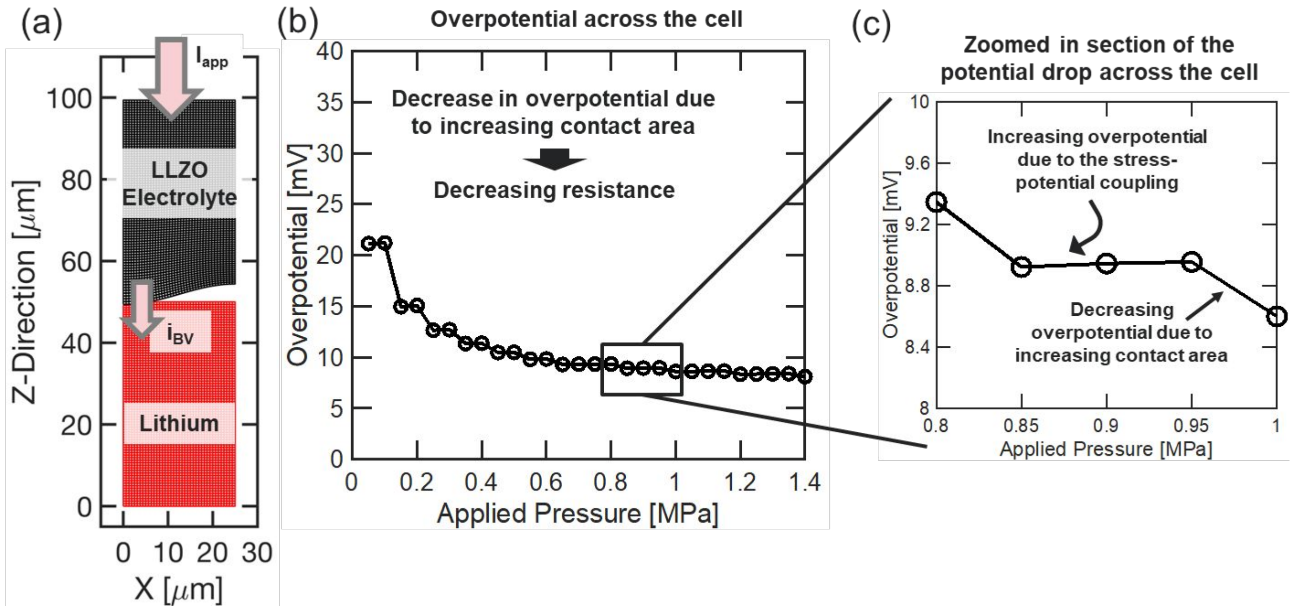

Figure S8. (a) Schematic of the computational mesh used to simulate the overpotential at a contact area of the lithium-LLZO interface with the LLZO solid electrolyte (black domain) and the lithium metal electrode (red region). (b) Overpotential plotted as a function of the applied external pressure. The overpotential generally decreases with the applied external pressure. However, at certain pressures, the overpotential increases due to stress-potential coupling, which is highlighted in the inset (c).

The deformed Li/LLZO computational domain with the presence of the 50 nm thick gold (Au) layer in between is shown in Figure S10(a). The external pressure is increased to 1.2 MPa to obtain the deformed computational domain. The Au domain is assumed to deform elastically during the application of the mechanical stress, because the yield strength of Au is more than 50 MPa, which is never reached within the Au layer under the application of such limited external pressure (~ 1.2 MPa). The Au layer is also in perfect contact with the LLZO solid electrolyte, and its contact with Li metal electrode improves with increasing external pressure. A zoomed in view of the Li/LLZO interface, with the 50 nm thick gold (Au) layer, is shown in Figure S10(b), which clearly indicates the good contact between the Au layer and LLZO.

For computationally predicting the electrochemical response of the Li/Au/LLZO system, Li from the LLZO is assumed to electrochemically alloy with Au, instead of depositing directly on Li metal. Electrons flowing through the Li electrode enters the Au layer and react with Li ions from LLZO according to the Butler-Volmer equation shown in Eq. (S1). The 50 nm thick gold (Au) layer is deposited on top of LLZO electrolyte before bringing it in contact with Li electrode, which results in extremely good contact between the Au and LLZO. Since the electrochemical reaction occurs between Au and LLZO, the electrochemically active surface area does not change with external pressure and always remains constant. External pressure increases the contact between the Li and Au, where transport of electrons take place. Since the electronic conductivity of both

the metals are extremely high (> 10 MS/m), and potential drop for transport of electrons from Li to Au is assumed to be zero (perfect interface), increasing the contact between the Li electrode and Au layer does not result in any appreciable change in the total overpotential associated with flowing current through the Li/Au/LLZO system. As a result, for Li/Au/LLZO system, the electrochemical resistance does not change significantly with increasing pressure.

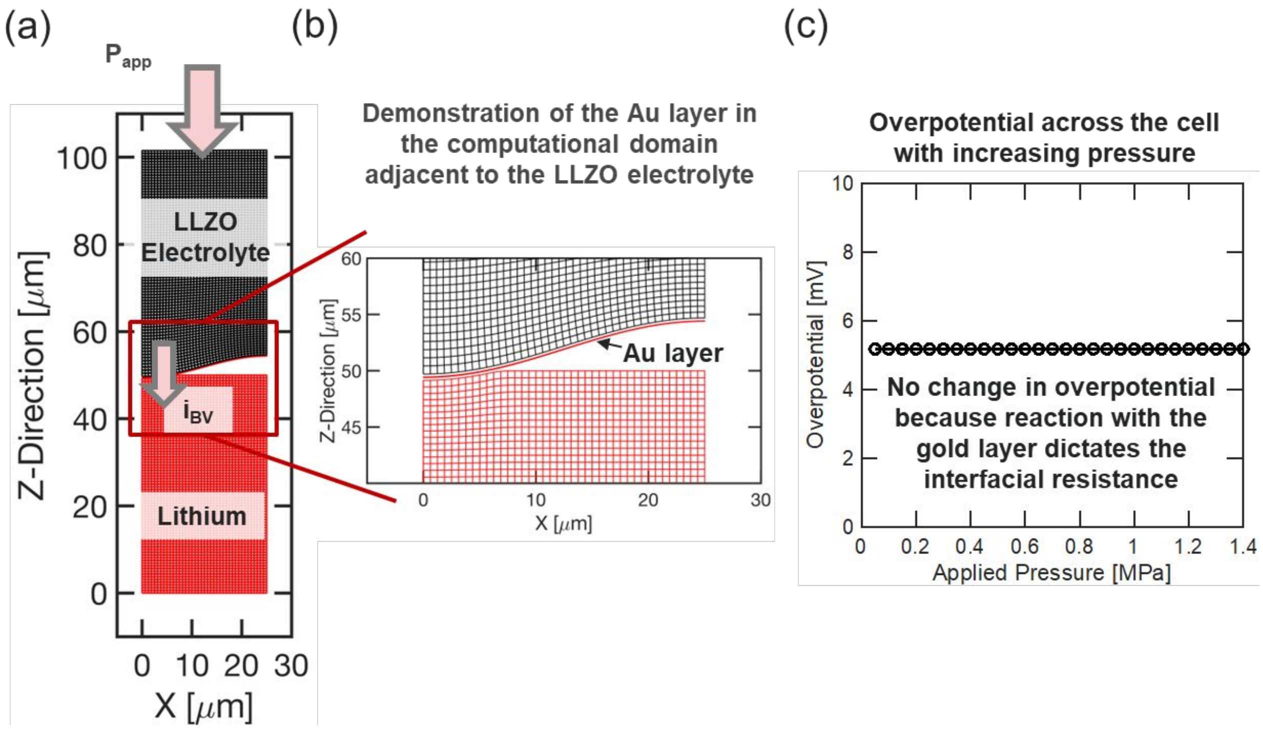

Figure S9. (a) Schematic of the computational mesh used to simulate the overpotential at a contact area of the lithium-LLZO interface with Li electrode (red) and gold (Au) deposited LLZO electrolyte (black) interface under an externally applied pressure, (b) zoomed-in section of the mesh showing the 50nm gold (Au) layer, (c) Overpotential plotted as a function of the applied external pressure. There is no change in the interface overpotential with pressure as the interface overpotential is dictated by the reaction of the gold layer. This observation is consistent with results from EIS.

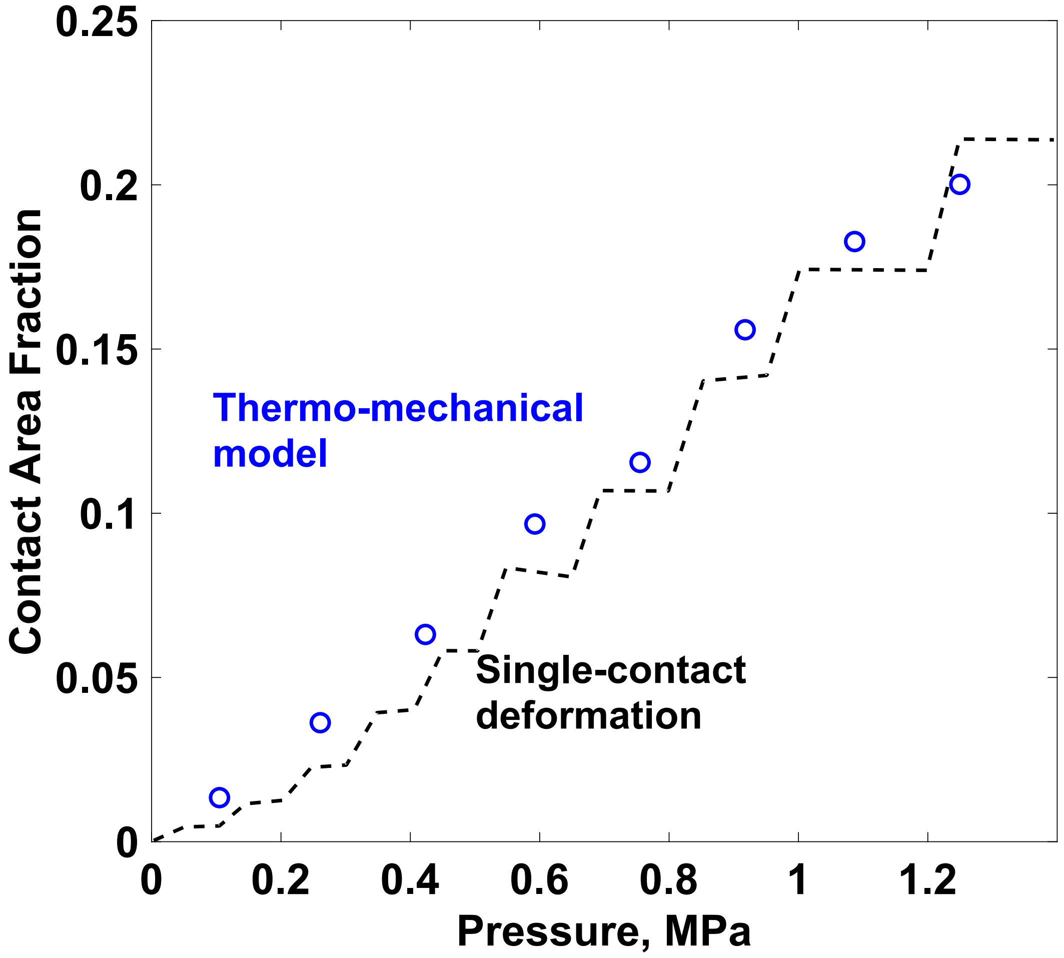

Figure S10. Contact area fraction for the lithium-LLZO contact calculated from the single-contact model used for simulating the interface overpotential compared with the contact area fraction obtained from Yovanovich's thermo-mechanical model[9] used for relating the interface morphology and the thermal contact resistance. The results from the two models are identical which shows that the two models are mechanically equivalent.

Table S2. List of governing equations (GE) used in the electrochemical computational analysis and relevant boundary conditions (BCs)

GEs and BCs	Lithium electrode	LLZO electrolyte
GE for charge balance	$\vec{\nabla} \cdot (\kappa_{\mathrm{Li}} \vec{\nabla} \phi_{\mathrm{Li}}) = 0$	$\vec{\nabla} \cdot (\kappa_{\mathrm{LLZO}} \vec{\nabla} \phi_{\mathrm{LLZO}}) = 0$
BC at top and bottom	$-\kappa_{\mathrm{Li}} \vec{\nabla} \phi_{\mathrm{Li}} \big\|_{\mathrm{y}=0} = I_{\mathrm{app}}$<br>$-\kappa_{\mathrm{Li}} \vec{\nabla} \phi_{\mathrm{Li}} \big\|_{\mathrm{Li\|LLZO}} = i_{\mathrm{BV}}$	$-(\kappa_{\mathrm{LLZO}} \vec{\nabla} \phi_{\mathrm{LLZO}}) \big\|_{\mathrm{Li\|LLZO}} = i_{\mathrm{BV}}$<br>$\phi_{\mathrm{LLZO}} \big\|_{\mathrm{y\sim top}} = 0.0$
GE for stress equilibrium	$\vec{\nabla} \cdot \bar{\bar{\sigma}}_{\mathrm{Li}} = 0$	$\vec{\nabla} \cdot \bar{\bar{\sigma}}_{\mathrm{LLZO}} = 0$
BC for stress equilibrium	$(u_{\mathrm{x}}, u_{\mathrm{y}}) \big\|_{\mathrm{y}=0} = 0$<br>$(u_{\mathrm{x}}, u_{\mathrm{y}}, f_{\mathrm{x}}, f_{\mathrm{y}}) \big\|_{\mathrm{Li\|LLZO}} \rightarrow$balanced	$f_{\mathrm{x}} \big\|_{\mathrm{y\sim top}} = 0$ and $f_{\mathrm{y}} \big\|_{\mathrm{y\sim top}} = P_{\mathrm{app}} \cdot Area$<br>$(u_{\mathrm{x}}, u_{\mathrm{y}}, f_{\mathrm{x}}, f_{\mathrm{y}}) \big\|_{\mathrm{Li\|LLZO}} \rightarrow$balanced
Elastic stress-strain relations	$\bar{\bar{\sigma}}_{\mathrm{Li}} = \bar{\bar{C}}^{\mathrm{e}}_{\mathrm{Li}} \bar{\bar{\epsilon}}_{\mathrm{Li}}$	$\bar{\bar{\sigma}}_{\mathrm{LLZO}} = \bar{\bar{C}}^{\mathrm{e}}_{\mathrm{LLZO}} \bar{\bar{\epsilon}}_{\mathrm{LLZO}}$
Strain elastic-plastic decomposition	$\bar{\bar{\epsilon}}_{\mathrm{Li}} = \bar{\bar{\epsilon}}^{\mathrm{e}}_{\mathrm{Li}} + \bar{\bar{\epsilon}}^{\mathrm{p}}_{\mathrm{Li}}$	$\bar{\bar{\epsilon}}_{\mathrm{LLZO}} = \bar{\bar{\epsilon}}^{\mathrm{e}}_{\mathrm{LLZO}} + \bar{\bar{\epsilon}}^{\mathrm{p}}_{\mathrm{LLZO}}$
Yield stress	$\sigma_{\mathrm{y,Li}} = \sigma_{\mathrm{y,Li,0}} + H_{\mathrm{Li}} \epsilon^{\mathrm{p}}_{\mathrm{eq,Li}}$	$\sigma_{\mathrm{y,LLZO}} = \sigma_{\mathrm{y,LLZO,0}} + H_{\mathrm{LLZO}} \epsilon^{\mathrm{p}}_{\mathrm{eq,LLZO}}$

Table S3. List of parameters used for the interface overpotential simulations

Name	Symbol	Unit	Value	Notes
Conductivity of LLZO[10]	κ_{LLZO}	S/m	10^{-2}	
Conductivity of Li[7]	κ_{Li}	S/m	1.1×10^{7}	
Universal gas constant	R	J/mol · K	8.314	
Temperature	T	K	300	
Faraday constant	F	C/mol	96485	
Reference exchange current density at Li/LLZO interface[11]	$i_{0,\mathrm{ref}}$	$\mathrm{A/m^2}$	100	
Shear modulus of Li[7]	G_{Li}	GPa	3.4	
Poisson's ratio of Li[7]	ν_{Li}	--	0.42	
Shear modulus of LLZO[12]	G_{LLZO}	GPa	52.7	
Poisson's ratio of LLZO[13]	ν_{LLZO}	--	0.33	
Yield strength of Li	$\sigma_{0,\mathrm{Li}}$	MPa	2.0	Assumed
Yield strength of LLZO	$\sigma_{0,\mathrm{LLZO}}$	MPa	∞	Elastic response

Supporting Information References

(1) Yi, E.; Shen, H.; Heywood, S.; Alvarado, J.; Parkinson, D. Y.; Chen, G.; Sofie, S. W.; Doeff, M. M. All-Solid-State Batteries Using Rationally Designed Garnet Electrolyte Frameworks. *ACS Appl. Energy Mater.* **2020**, *3* (1), 170–175. https://doi.org/10.1021/acsaem.9b02101.

(2) Zeng, Y.; Chalise, D.; Fu, Y.; Battaglia, V.; Lubner, S. D.; Prasher, R. S. Operando Spatial Mapping of Lithium Concentration Using Thermal-Wave Sensing. *Joule* **2021**, *5*, 1–16. https://doi.org/10.1016/j.joule.2021.06.008.

(3) Lubner, S. D.; Kaur, S.; Fu, Y.; Battaglia, V.; Prasher, R. S. Identification and Characterization of the Dominant Thermal Resistance in Lithium-Ion Batteries Using *Operando* 3-Omega Sensors. *J. Appl. Phys.* **2020**, *127* (10), 105104. https://doi.org/10.1063/1.5134459.

(4) *Parylene Properties | Specialty Coating Systems*.

(5) Lide, D. R. *CRC Handbook of Chemistry and Physics, 90th Edition (CD-ROM Version 2010)*; 2009; Vol. 131.

(6) Ho, C. Y.; Powell, R. W.; Liley, P. E. Thermal Conductivity of the Elements. *J. Phys. Chem. Ref. Data 1* **1972**, *279*. https://doi.org/https://doi.org/10.1063/1.3253100 Published.

(7) Monroe, C.; Newman, J. The Impact of Elastic Deformation on Deposition Kinetics at Lithium/Polymer Interfaces. *J. Electrochem. Soc.* **2005**, *152* (2), A396. https://doi.org/10.1149/1.1850854.

(8) Monroe, C.; Newman, J. The Effect of Interfacial Deformation on Electrodeposition Kinetics. *J. Electrochem. Soc.* **2004**, *151* (6), A880. https://doi.org/10.1149/1.1710893.

(9) Sridhar, M. R.; Yovanovich, M. M. Elastoplastic Contact Conductance Model for Isotropic Conforming Rough Surfaces and Comparison With Experiments. *J. Heat Transfer* **1996**, *118*. https://doi.org/10.1115/1.2824241.

(10) Krauskopf, T.; Hartmann, H.; Zeier, W. G.; Janek, J. Toward a Fundamental Understanding of the Lithium Metal Anode in Solid-State Batteries - An Electrochemo-Mechanical Study on the Garnet-Type Solid Electrolyte Li 6.25 Al 0.25 La 3 Zr 2 O 12. *ACS Appl. Mater. Interfaces* **2019**, *11* (15), 14463–14477. https://doi.org/10.1021/acsami.9b02537.

(11) Sharafi, A.; Kazyak, E.; Davis, A. L.; Yu, S.; Thompson, T.; Siegel, D. J.; Dasgupta, N. P.; Sakamoto, J. Surface Chemistry Mechanism of Ultra-Low Interfacial Resistance in the Solid-State Electrolyte Li7La3Zr2O12. *Chem. Mater.* **2017**, *29* (18), 7961–7968. https://doi.org/10.1021/acs.chemmater.7b03002.

(12) Barai, P.; Ngo, A. T.; Narayanan, B.; Higa, K.; Curtiss, L. A.; Srinivasan, V. The Role of Local Inhomogeneities on Dendrite Growth in LLZO-Based Solid Electrolytes. *J. Electrochem. Soc.* **2020**, *167* (10), 100537. https://doi.org/10.1149/1945-7111/ab9b08.

(13) Yu, S.; Schmidt, R. D.; Garcia-Mendez, R.; Herbert, E.; Dudney, N. J.; Wolfenstine, J. B.; Sakamoto, J.; Siegel, D. J. Elastic Properties of the Solid Electrolyte Li7La3Zr2O12

(LLZO). *Chem. Mater.* **2016**, *28* (1), 197–206. https://doi.org/10.1021/acs.chemmater.5b03854.
